# A Dietary Oxysterol, 7-Ketocholesterol, Exacerbates Imiquimod-Induced Psoriasis-like Dermatitis in Steatohepatitic Mice

**DOI:** 10.3390/ijms232415855

**Published:** 2022-12-13

**Authors:** Ayami Saga, Masahiro Koseki, Kotaro Kanno, Jiuyang Chang, Tomoaki Higo, Daisuke Okuzaki, Takeshi Okada, Hiroyasu Inui, Masumi Asaji, Katsunao Tanaka, Takashi Omatsu, Sae Nishihara, Yinghong Zhu, Kaori Ito, Hiroaki Hattori, Ikuyo Ichi, Yoshihiro Kamada, Masafumi Ono, Toshiji Saibara, Tohru Ohama, Shungo Hikoso, Makoto Nishida, Shizuya Yamashita, Yasushi Sakata

**Affiliations:** 1 Department of Cardiovascular Medicine, Osaka University Graduate School of Medicine, Osaka 565-0871, Japan; 2 Genome Information Research Center, Research Institute for Microbial Diseases, Osaka University, Osaka 565-0871, Japan; 3 Department of Advanced Medical Technology and Development, BML, Inc., Kawagoe TX 79608, Japan; 4 Institute for Human Life Innovation, Ochanomizu University, Tokyo 112-8610, Japan; 5 Department of Advanced Metabolic Hepatology, Osaka University Graduate School of Medicine, Osaka 565-0871, Japan; 6 Division of Innovative Medicine for Hepatobiliary & Pancreatology, Faculty of Medicine, Kagawa University, Kagawa 760-0016, Japan; 7 Department of Gastroenterology and Hepatology, Kochi Medical School, Kochi 783-8505, Japan; 8 Health Care Division, Health and Counseling Center, Osaka University, Osaka 565-0871, Japan; 9 Rinku General Medical Center, Osaka 598-8577, Japan

**Keywords:** 7-ketocholesterol, psoriasis, steatohepatitis, interleukin-17, tumor necrosis factor-α

## Abstract

Patients with psoriasis are at a higher risk of developing nonalcoholic fatty liver disease. We previously identified an oxidized derivative of cholesterol, 7-ketocholesterol (7KC), in diet-induced steatohepatitic mice. Here, we investigated whether 7KC exacerbates psoriasis-like dermatitis by accelerating steatohepatitis in mice. A high-fat/high-cholesterol/high-sucrose/bile salt diet (nonalcoholic steatohepatitis (NASH) diet) with or without 0.0125% 7KC was fed to C57BL/6 mice (7KC or control group) for three weeks to induce steatohepatitis. A 5% imiquimod cream was then applied to the ears and dorsal skin for four days to induce psoriasis-like dermatitis. Hepatic lipid accumulation and inflammatory cell infiltration were exacerbated in the 7KC group compared with the control group after three weeks. Serum tumor necrosis factor-α (TNF-α) levels were also elevated in the 7KC group (108.5 ± 9.8 vs. 83.1 ± 13.1 pg/mL, *p* < 0.005). Imiquimod cream increased the psoriasis area severity index (PASI) score in mice in the 7KC group (9.14 ± 0.75 vs. 5.17 ± 1.17, *p* < 0.0001). Additionally, *Tnfa*, *Il23a*, *Il17a*, and *Il22* mRNA levels in the dorsal lesion were significantly upregulated. Finally, Th17 cell differentiation and the TNF signaling pathway were enhanced in the dorsal lesions and liver of mice in the 7KC group. These data suggest that steatohepatitis and psoriasis are linked by a potent, diet-related factor.

## 1. Introduction

Psoriasis is a chronic inflammatory skin condition characterized by hyperproliferation of epidermal cells and is associated with lifestyle-related diseases [1,2,3]. Patients with psoriasis are at a higher risk of developing myocardial infarction [4]. The disease is also associated with metabolic syndrome and obesity [5,6]. In addition, an increase in the incidence of nonalcoholic fatty liver disease (NAFLD) has been observed among patients with psoriasis [7,8,9,10]. On the other hand, NAFLD may be an important factor underlying cardiovascular diseases, separate from obesity and metabolic syndrome [11]. The mechanism underlying psoriasis and chronic inflammatory diseases such as atherosclerosis has been explained by the involvement of T-cell activation and/or inflammatory cytokines such as tumor necrosis factor-α (TNF-α), interleukin (IL)-17, and IL-23 [1,2,12,13,14,15]. However, other factors between psoriasis and NAFLD, e.g., nutrition-related factors, have not been fully investigated.

Oxidative derivatives of cholesterol are categorized into endogenous and exogenous oxysterols [16]. Endogenous oxysterols such as 22-hydroxycholesterol or 27-hydroxycholesterol are produced by endogenous enzymes when intracellular cholesterol is in excess, and act as a ligand for the nuclear receptor liver X receptor. In contrast, exogenous sterols are produced via the natural oxidation of cholesterol in the diet or by cooking cholesterol-containing foods [16,17]. One of these, 7-ketocholesterol (7KC), is an exogenously oxidized sterol that has been detected in the blood and atherosclerotic lesions of hypercholesterolemic patients [18,19]. Song et al. [20] found that patients with higher 7KC levels in their plasma had a higher incidence of cardiovascular events. Several studies have revealed that butter, yogurt, and especially processed meats such as ham and sausage, are relatively rich in 7KC (7.3, 4, 12.04, and 8.9 μg/g, respectively) [16]. We hypothesized that dietary 7KC might accelerate steatohepatitis and examined whether dietary 7KC might promote hepatic lipid accumulation and inflammation in obese mouse models (ob/ob mice). We previously demonstrated that a small quantity of dietary 7KC accelerates hepatic steatosis and inflammation in obese mouse models [21]. Therefore, in this study, we explored whether a high-fat diet containing 7KC could exacerbate steatohepatitis and enhance imiquimod (IMQ)-induced psoriasis-like dermatitis in wild type mice.

## 2. Results

### 2.1. Dietary 7KC Promoted Hepatic Steatosis and Inflammation in C57BL/6 Mice

**Figure 1:**
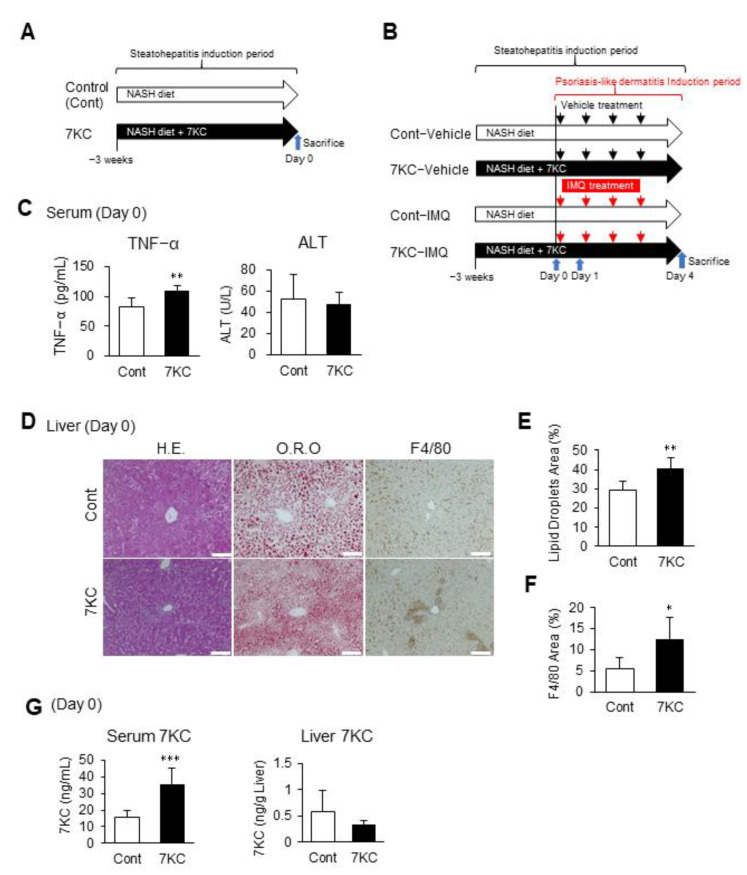
The 7KC-containing high-fat/high-cholesterol/high-sucrose/bile salt diet exacerbated steatohepatitis in C57BL/6 mice. (**A**) Experimental protocol before induction of psoriasis-like dermatitis. Samples were harvested on Day 0. (**B**) Experimental protocol after induction of psoriasis-like dermatitis. Samples were harvested on Day 1 and Day 4. (**C**) TNF-α and alanine aminotransferase (ALT) in serum on Day 0. *n* = 6, Control; *n* = 6, 7KC. (**D**) Hematoxylin and eosin (H.E.) staining, oil red O (O.R.O) staining, and F4/80 staining of liver samples on Day 0. Scale bar: 100 μm. (**E**) Areas with oil red O staining in the liver. *n* = 6, Control; *n* = 6, 7KC. (**F**) Areas with F4/80 staining in the liver. *n* = 6, Control; *n* = 6, 7KC. (**G**) 7KC concentrations in serum and liver of mice on Day 0. *n* = 5, Control; *n* = 5, 7KC. Results are presented as mean ± SD, and *p* values were calculated using Student’s *t*-test. * *p* < 0.05, ** *p* < 0.01, and *** *p* < 0.001, Control vs. 7KC.

In our previous study using obese and diabetic mice, we demonstrated that a high-fat diet containing 0.01% 7KC accelerated hepatic steatosis and inflammation [21]. We also previously demonstrated that a high-fat/high-cholesterol/bile salt diet, called the NASH diet, induces steatohepatitis and cardiomyopathy in male C57BL/6 mice [22]. Therefore, in the current study, we tested whether preconditioning with the 7KC-containing NASH diet might exacerbate steatohepatitis and promote imiquimod (IMQ)-induced psoriasis-like dermatitis in male C57BL/6 mice. To do this, we fed eight-week-old C57BL/6 mice the NASH diet with or without 0.0125% 7KC (7KC diet/NASH diet) for three weeks. We analyzed hepatic phenotypes on Day 0 (Figure 1A). Body and liver weights did not vary between the control and 7KC groups. However, spleen weights significantly increased in the 7KC group, indicating underlying inflammation (Figure S1A,B). Serum TNF-α levels were also significantly increased in the 7KC group (83.1 ± 13.1 vs. 108.5 ± 9.8 pg/mL, *p* < 0.005; Figure 1C). However, alanine aminotransferase (ALT), triglyceride (TG), total cholesterol (TC), high-density lipoprotein-cholesterol (HDL-C), and non-HDL-C levels in the serum did not vary between the control and 7KC groups (Figure 1C and Figure S1C). Along with these subacute changes, hepatic pathohistological analysis showed an increase in oil-red O-positive lipid droplets and an increase in F4/80 positive inflammatory-cell infiltration in the 7KC group compared with the control group (Figure 1D–F). To confirm the effect of 7KC, its concentrations in the serum and liver were measured using LC/MS–MS on Day 0. The concentration of 7KC increased significantly in the serum of mice in the 7KC group compared with that of mice in the control group (15.5 ± 4.1 vs. 35.8 ± 9.2 ng/µL, *p* < 0.001; Figure 1G). However, no significant differences were observed in the livers of mice in the two groups (Figure 1G).

### 2.2. 7KC Upregulated Th17 Cell Differentiation and the TNF Signaling Pathway in the Liver

**Figure 2:**
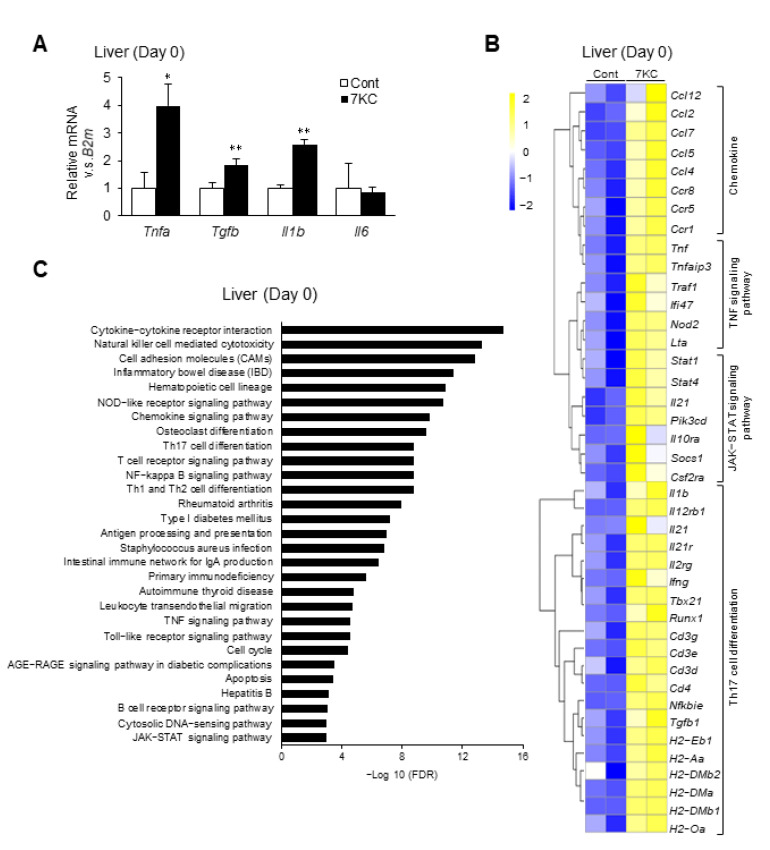
The 7KC-containing diet enhanced inflammatory responses in the liver. (**A**) mRNA expression of inflammatory genes was examined on Day 0 using quantitative PCR. Mean expression in the control group, obtained by correcting each CT value with the respective housekeeping gene, was used as a control (one) to indicate the relative expression. Results are presented as mean ± SD, and *p* values were calculated using Student’s *t*-test. * *p* < 0.05, and ** *p* < 0.01, Control vs. 7KC. *n* = 3, Control; *n* = 3, 7KC. (**B**) RNA sequence analysis of liver samples collected on Day 0. Heatmaps of genes associated with chemokines, TNF signaling pathway, JAK-STAT signaling pathway, and Th17 cell differentiation in the liver were identified. (**C**) Analysis of pathways in the liver on Day 0. Pathways with upregulated expression in the 7KC versus Control group are shown.

To confirm hepatic inflammatory responses, we performed a quantitative polymerase chain reaction (RT–qPCR) and RNA sequence analysis of liver samples collected on Day 0. *Tnfa* expression was approximately three-fold higher in the 7KC group than the control group (Figure 2A). *Tgfb* and *Il1b* expression were also significantly higher in the 7KC group than in the control group (Figure 2A). Furthermore, RNA sequence analysis revealed that the chemokine, TNF signaling, JAK-STAT signaling, and Th17 cell differentiation pathways were markedly upregulated in the 7KC group (Figure 2B,C). These data are consistent with previous reports that showed that *Tgfb* and *Il1b* are involved in Th17 cell differentiation [23]. RNA-sequence analysis also revealed that ATP binding cassette subfamily G member 1 (ABCG1), a 7KC transporter [24], was upregulated in 7KC-fed mice—indicating that up-regulated ABCG1 might suppress the accumulation of 7KC in liver (Figure S2). Collectively, adding 0.0125% of 7KC to the NASH diet promoted hepatic inflammation—especially in the TNF signaling pathway—in wild type mice on Day 0.

### 2.3. 7KC-Induced Steatohepatitis Enhanced the Proliferation of Dorsal Epidermal Cells

**Figure 3:**
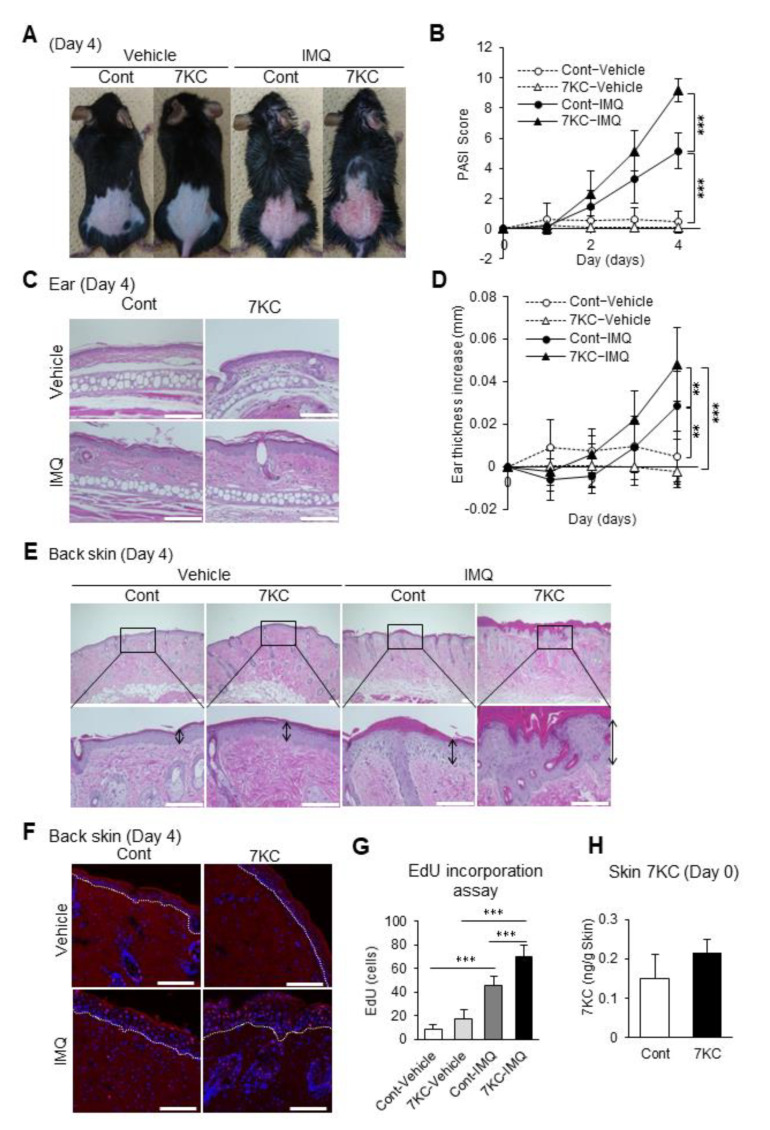
7KC-induced steatohepatitis exacerbated psoriasis-like dermatitis. Mice were fed a NASH or 7KC diet for three weeks, and then received imiquimod (IMQ) cream or hydrophilic cream (vehicle). (**A**) Representative photographs of mice on Day 4. (**B**) Time course of the psoriasis area severity index (PASI) scores after induction. *n* = 9, Cont-Vehicle; *n* = 9, 7KC-Vehicle; *n* = 6, Cont-IMQ; *n* = 6, 7KC-IMQ. (**C**) Hematoxylin and eosin (H.E.) staining of the ears. (**D**) Time course of the change in ear thickness after induction. *n* = 9, Cont-Vehicle; *n* = 9, 7KC-Vehicle; *n* = 12, Cont-IMQ; *n* = 13, 7KC-IMQ. (**E**) H.E. staining of the back skin on Day 4. (**F**) EdU incorporation assay of the back skin on Day 4. (**G**) Count of EdU-positive cells on Day 4. *n* = 5, Cont-Vehicle; *n* = 5, 7KC-Vehicle; *n* = 6, Cont-IMQ; *n* = 7, 7KC-IMQ. Scale bar: 100 μm; (**H**) 7KC concentration in the skin on Day 0. *n* = 5, Control; *n* = 5, 7KC. Results are presented as mean ± SD, and *p* values were calculated using one-way ANOVA with Tukey’s post hoc test (Figure 3B,D,G) and Student’s *t*-test (Figure 3H). ** *p* < 0.01, and *** *p* < 0.001, Cont-Vehicle vs. Cont-IMQ or Cont-Vehicle vs. 7KC-IMQ or Cont-IMQ vs. 7KC-IMQ or Control vs. 7KC. Scale bar: 100 μm.

Next, we examined whether exacerbating steatohepatitis using 7KC enhanced imiquimod-induced psoriasis-like dermatitis. After feeding mice on the NASH or 7KC diets for three weeks, control cream (vehicle) or 5% imiquimod cream (IMQ) was applied daily for four days to the right ear and dorsal skin (Figure 1B). In the IMQ group, mice on the 7KC diet showed psoriasis-like dermatitis, accompanied by more severe erythema and desquamation compared with mice on the NASH diet (Figure 3A). These phenotypes were not observed in the vehicle-treated group (Figure 3A). The degree of erythema and desquamation was quantified by calculating the psoriasis area severity index (PASI) score based on the condition of psoriasis-like dermatitis caused by erythema, desquamation, and thickening [25]. Compared with mice in the vehicle group fed with the NASH diet (Cont-Vehicle), the IMQ group fed with the NASH diet (Cont-IMQ) showed a marked increase in PASI scores on Day 4, suggesting that IMQ treatment successfully induced psoriasis-like dermatitis (5.17 ± 1.17 vs. 0.44 ± 0.73; *p* < 0.0001; Figure 3B). Importantly, the PASI score was significantly higher in the IMQ group fed with the 7KC diet (7KC-IMQ) compared with the Cont-IMQ group (9.14 ± 0.75 vs. 5.17 ± 1.17; *p* < 0.0001; Figure 3B). A histological analysis of skin from the ears was also performed (Figure 3C). Consistent with the PASI score, the auricular thickness of the mice in the 7KC-IMQ group was significantly greater than that in the Cont-IMQ group on Day 4 (0.029 ± 0.016 mm vs. 0.048 ± 0.017 mm, *p* < 0.001; Figure 3D). In contrast, there were no differences between mice in the 7KC-Vehicle and the Cont-Vehicle groups (Figure 3D). In the dorsal lesion, the epidermal thickness was also markedly greater in the 7KC-IMQ group (Figure 3E). Next, we examined the proliferation process using the 5-ethynil-2’-deoxyuridine (EdU) proliferation assay, which allows the detection of dividing and/or proliferating cells. Mice were injected with EdU on Day 4, sacrificed, and the EdU assay was performed on the dorsal lesions. The number of EdU-positive cells in the epidermal layer was significantly higher in the 7KC-IMQ group than in the Cont-IMQ group, suggesting that 7KC promotes the proliferation of the basal layer of the dorsal lesion (Figure 3F,G). The direct effect of the accumulation of 7KC was assessed by measuring the concentration of 7KC in the dorsal lesion using LC/MS–MS prior to IMQ treatment (Day 0). There was no significant difference between the control and 7KC groups (Figure 3H), suggesting that the 7KC-inducing inflammatory response in the liver and serum might promote psoriasis-like dermatitis following the application of IMQ.

### 2.4. TNF- and IL-17A-Related Signaling Pathways Were Enhanced in the Dorsal Skin of Mice in the 7KC-IMQ Group

**Figure 4:**
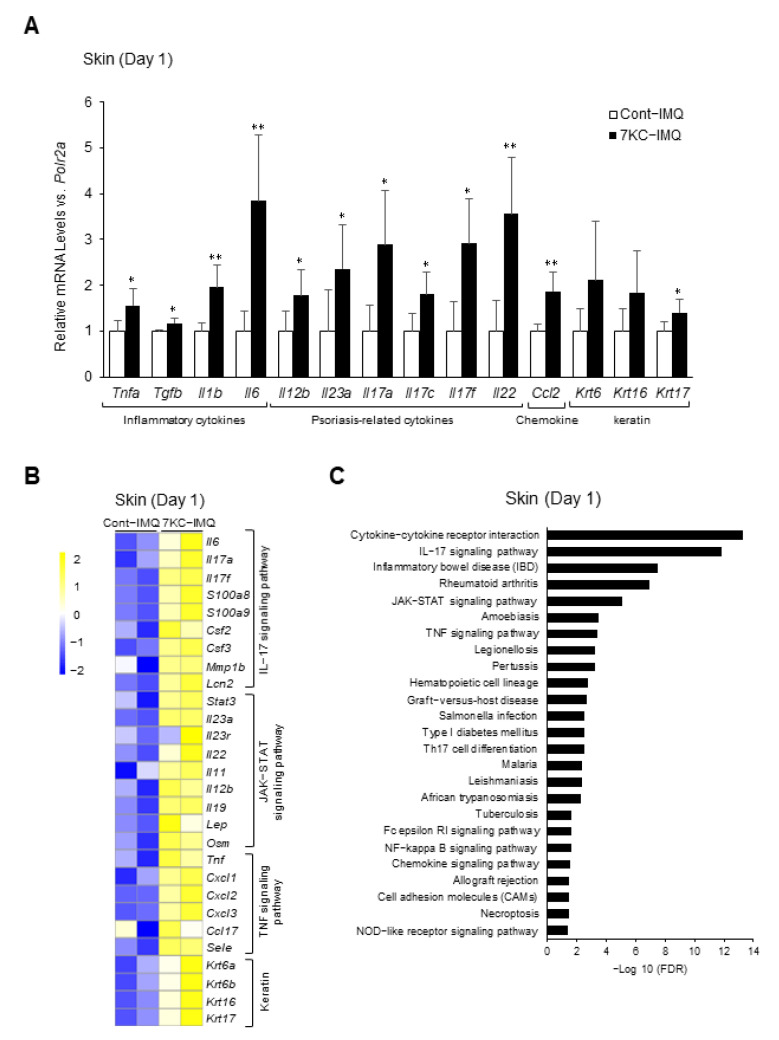
7KC-induced steatohepatitis promoted the TNF- and IL-17 signaling pathways in the skin. Mice were fed a NASH diet or 7KC diet for three weeks, and then received imiquimod (IMQ) cream or hydrophilic cream (vehicle). (**A**) mRNA expression was examined using quantitative PCR on Day 1. Mean expression in the Control group, obtained by correcting each CT value with the respective housekeeping gene, was used as a control (one) to indicate the relative expression. Results are presented as mean ± SD, and *p*-values were calculated using Student’s *t*-test. * *p* < 0.05, and ** *p* < 0.01, Cont vs. 7KC. *n* = 5, Cont; *n* = 5, 7KC. (**B**) RNA sequence analysis of skin samples collected on Day 1. Heatmaps of genes associated with chemokines, TNF signaling pathway, JAK-STAT signaling pathway, and Th17 cell differentiation in the skin were generated. Heatmaps of genes associated with chemokines, TNF, JAK-STAT, and IL-17 signaling pathways, and keratin in the skin. (**C**) Pathways whose expressions were upregulated in the 7KC group versus the control group are listed.

The mRNA expression of psoriasis-related cytokines at the site of IMQ-induced psoriasis-like dermatitis peaked 1–3 days after induction, before decreasing [26]. Therefore, we performed a quantitative PCR and RNA sequence analysis of samples collected on Day 1 to confirm the inflammatory response in the dorsal lesions. The expression of inflammatory cytokines such as *Tnfa*, *Tgfb*, *Il6*, Il12b, *Il23a*, *Il17a*, Il17c, *Il17f*, *Il22*, and *Il1b* was significantly upregulated in the 7KC-IMQ group (Figure 4A). The mRNA levels of inflammasome-related *Nlrp3* and the macrophage-associated chemokine *Ccl2* were also significantly higher in the 7KC-IMQ group (Figure 4A). The expression of *Krt17*, a marker of keratinocyte proliferation [27], was also significantly upregulated in the 7KC-IMQ group (Figure 4A). An RNA sequence analysis of dorsal lesion samples revealed that the IL-17A, JAK-STAT, and TNF signaling pathways and Th17 differentiation were enhanced in the 7KC-IMQ group (Figure 4B,C). The JAK-STAT signaling pathway is reportedly promoted during psoriasis [28,29]. Therefore, these results are consistent with the worsening of psoriasis-like dermatitis in the 7KC-IMQ group.

### 2.5. Inflammatory Responses Were Exacerbated in the 7KC-IMQ Group following IMQ Treatment

**Figure 5:**
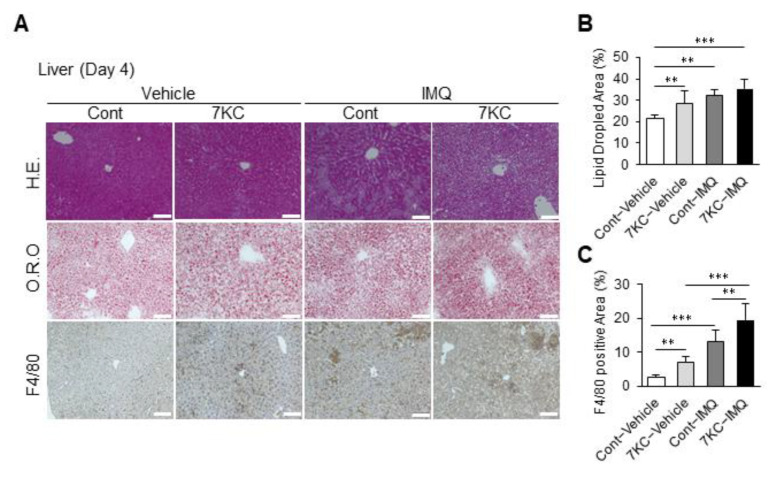
Psoriasis-like dermatitis aggravated by 7KC further accelerated steatohepatitis. (**A**) Hematoxylin and eosin (H.E.) staining, oil red O (O.R.O) staining, and F4/80 staining of liver on Day 4. (**B**) Areas with oil red O staining in the liver. *n* = 5, Cont-Vehicle; *n* = 6, 7KC-Vehicle; *n* = 6, Cont-IMQ; *n* = 6, 7KC-IMQ. (**C**) Areas with F4/80 staining in the liver. *n* = 6, Cont-Vehicle; *n* = 6, 7KC-Vehicle; *n* = 6, Cont-IMQ; *n* = 6, 7KC-IMQ. Results are presented as mean ± SD, and *p* values were calculated using one-way ANOVA with Tukey’s post hoc test. ** *p* < 0.01, and *** *p* < 0.001, Cont-Vehicle vs. Cont-IMQ, Cont-Vehicle vs. 7KC-IMQ, Cont-IMQ vs. 7KC-IMQ or 7KC-Vehicle vs. 7KC-IMQ. Scale bar: 100 μm.

Lipid droplet and macrophage infiltration in the liver was measured on Day 4 to examine the effects of psoriasis-like dermatitis induction on steatohepatitis. Both lipid deposition and macrophage infiltration were significantly increased in the 7KC-Vehicle group compared with the Cont-Vehicle group, similar to what was observed on Day 0 (Figure 1D–F and Figure 5A–C). There was no significant difference in lipid droplets in the 7KC-IMQ and Cont-IMQ groups, but macrophage infiltration was strongly enhanced (Figure 5A–C). These inflammatory responses in the liver may suggest that worsening psoriasis-like dermatitis might further aggravate steatohepatitis in the presence of 7KC. 

## 3. Discussion

Psoriasis and NAFLD are inflammatory disorders. However, a common genetic background has not been reported, indicating the involvement of lifestyle-related factors. We hypothesized that an oxidized cholesterol molecule, 7KC, might be one of these lifestyle-related factors; 7KC exerts a pro-atherosclerotic effect on human monocytes by promoting the activation of NF-κB and increasing the production of inflammatory cytokines [30,31,32]. We previously studied obese mouse models and found that fat accumulation and inflammatory cell infiltration increased in the liver, with steatohepatitis being exacerbated when the obese mice were fed a high-fat diet containing 1% cholesterol and 0.01% 7KC [21]. In the current study, wild type mice fed a high-fat/high-cholesterol/high-sucrose/bile salt diet containing 0.0125% 7KC (7KC diet) showed a significant increase in serum 7KC concentrations and accelerated hepatic steatosis and inflammatory cell infiltration. However, 7KC levels in the skin did not significantly increase (Figure 2F), suggesting that inflammatory responses in the liver or blood might have an impact on the initiation and progression of psoriasis-like dermatitis. Importantly, mice fed with 7KC and treated with IMQ had higher levels of F4/80-positive inflammatory cell infiltration in the liver compared with mice fed on 7KC treated with the vehicle, indicating the existence of inflammatory feedback from the skin to the liver (Figure 5A,C).

**Figure 6:**
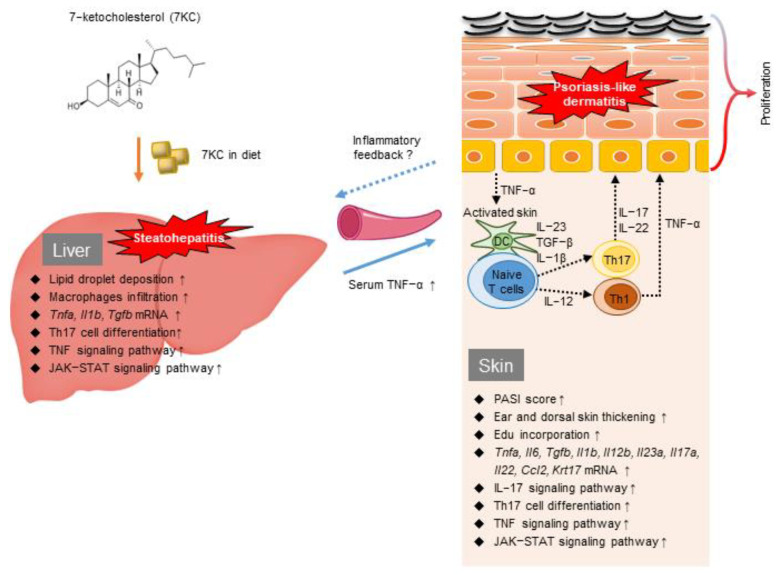
The putative mechanism linking steatohepatitis and psoriasis-like dermatitis stimulated by 7KC and a graphical summary of the current study.

The putative mechanisms linking steatohepatitis and psoriasis-like dermatitis are summarized in Figure 6. We consider that diet-derived 7KC in the blood and liver stimulated inflammatory cells and TNF-α and IL-1b secretion, leading to Th17 cell activation. Accordingly, *Tnfa*, *Il1b*, and *Tgfb* mRNA expression was upregulated in the livers of mice in the 7KC group (Figure 2A). An RNA sequence analysis of liver samples revealed the activation of TNF signaling, Th17 cell differentiation, and JAK-STAT signaling pathways (Figure 2B,C). In the 7KC group, serum TNF-α was increased and spleen weight was increased, suggesting that steatohepatitis induced systemic inflammation (Figure 1C and Figure S1C). Therefore, we have considered that steatohepatitis may exacerbate IMQ-induced psoriasis-like dermatitis by elevating TNF-α through systemic inflammation. These results are consistent with those of our previous study, which was based on an obese mouse model [21]. Given that a higher frequency of Th17 cells was previously reported in the livers of nonalcoholic steatohepatitis patients [33], we used FACS to investigate whether the Th17 cell fraction was higher in the blood of this mouse model. No significant change was observed in this model. We assumed that Th17 cells are localized in the liver and skin. Conversely, an RNA sequence analysis of dorsal skin samples revealed that *Il23a*, *Il17a*, and *Il22* expression was upregulated on Day 1 in the 7KC-IMQ group compared with the Cont-IMQ group (Figure 4A), indicating that the JAK-STAT and IL-17 signaling pathways and Th17 cell differentiation were activated in the 7KC-IMQ group compared with the Cont-IMQ group (Figure 4B,C). These results suggest that pre-feeding with 7KC diets induces greater dynamic inflammatory responses in the skin, although previous experiments using IMQ have demonstrated a dramatic induction of inflammation [26,34,35]. Interestingly, the 7KC-mediated promotion of TNF-α secretion from inflammatory cells in the skin may trigger an inflammatory loop between the liver and skin, contributing to the promotion of psoriasis-like dermatitis. A couple of studies indicated that oxysterols could bind to oxysterol-binding proteins (OSBP) and circulate systemically, acting directly on IMQ skin lesions. If 7KC may bind to OSBP and be transported by OSBP, then OSBP might be a potential therapeutic target against both steatohepatitis and psoriasis.

A limitation of this study was the short duration of the IMQ application. The period over which IMQ was administered was insufficient to observe its accumulation in the skin; it was also too short to assess hepatic fibrosis. Although Vasseur P. et al. nicely demonstrated that IMQ-induced dermatitis accelerated hepatis fibrosis in a 9-week model [36], Yokogawa M. et al. reported that administering IMQ for more than one month causes systemic lupus erythematosus-like dermatitis and might be not suitable for long-term use [37]. To study the long-term effects of psoriasis-like dermatitis on steatohepatitis and hepatic fibrosis, a genetically modified psoriasis model such as the K5.STAT3C transgenic mouse was developed by Sano et al. [38]. In future studies, we will use this model to clarify the relationship between psoriasis, steatohepatitis, and cardiovascular disease in order to reveal additional potential mechanisms, including those involving exosomes and microRNAs.

## 4. Materials and Methods

### 4.1. Animals, Diets, and Induction of Psoriasis Using IMQ

Wild type male C57BL/6 mice were obtained from CLEA Japan, Inc. (Tokyo, Japan) and housed in a temperature- and humidity-controlled facility with a 12 h light/dark cycle. The 7KC was obtained from Sigma-Aldrich (C2394, St. Louis, MO, USA). Two diets containing bile and high-fat/high-cholesterol/high-sucrose and bile salt (20% casein, 50% sucrose, 15% cocoa butter, 1.25% cholesterol, 0.5% cholate; NASH diet), containing 0.0125% 7KC (7KC group) or no 7KC (control group), were prepared by the Oriental Yeast Co., Ltd. (Chiba, Japan) and administered to the mice for three weeks. This was followed by the application of a 5% IMQ cream (5% Beselna Cream, Mochida Pharmaceutical Co., LTD, Tokyo, Japan) to the right ear (12.5 mg/day) and on the dorsal skin (62.5 mg/day) for four days. A hydrophilic cream (Nikko Pharmaceutical Co., LTD, Gifu, Japan) was used as the vehicle (Figure 1A,B). The thickness of the auricle was measured using a micrometer (Mitutoyo Corporation, Kanagawa, Japan) and was set to 0 before applying the IMQ or hydrophilic cream.

Mice were sacrificed by anaesthetization using an intraperitoneal injection of medetomidine (0.3 mg/kg), midazolam (4 mg/kg), and butorphanol (5 mg/kg). Adequate anesthesia was maintained by monitoring the respiration rate and lack of response to paw pinching. After the study, all anesthetized animals were euthanized via cervical dislocation. 

### 4.2. Psoriasis Area Severity Index (PASI)

Inflammation in the skin of mice was assessed using scores adapted from the clinical psoriasis area and severity index. Erythema, desquamation, and thickening were quantified as 0: none, 1: mild, 2: moderate, 3: high, or 4: extremely high. The total PASI score (0–12) was calculated as described by Fredriksson et al. [25,26].

### 4.3. Biochemical Analyses

Serum ALT, TC, HDL-C, and TG levels were measured using enzymatic methods (Fujifilm, Tokyo, Japan). Non-HDL-C levels were calculated as TC minus HDL-C levels. Plasma lipoprotein levels were measured after fasting the mice for 4 h. Serum TNF-α, IL-17, and IL-22 levels were measured using the Quantikine^®^ ELISA Mouse TNF-α Immunoassay, Quantikine^®^ ELISA Mouse IL-17 Immunoassay, and Quantikine^®^ ELISA Mouse IL-22 Immunoassay (MTA00B, M1700 and M2200, R&D systems, Minneapolis, MN, USA), respectively, in accordance with the manufacturer’s instructions. 

### 4.4. Measuring 7KC Levels in Serum and Tissues

Lipids were extracted from the serum, liver, and skin using the Folch method, and the concentration of 7KC in these samples was measured. Serum (20 μL) was saponified with potassium hydroxide and then mixed with magnesium sulfate and tert-butyl methyl ether. After the ether layer was extracted, it was dried under the N_2_ gas stream, dissolved with 40 μL of isopropanol, and used to measure the concentration of 7KC using LC/MS–MS. For liver and skin tissues, 30 mg of sample was extracted using the Folch method and saponified with potassium hydroxide. The lipid layer was extracted by the same method for serum samples, and the concentration of 7KC was measured using LC/MS–MS.

### 4.5. Histological and Immunohistochemical Analyses

Liver and skin tissues were fixed with 4% PFA to prepare paraffin sections. Paraffin-embedded sections were stained with hematoxylin and eosin (200108, Muto Pure Chemicals, Tokyo, Japan). Frozen sections were stained with Oil Red O (M3G0644; Nacalai Tesque, Kyoto, Japan) to detect lipids. Macrophages were detected using F4/80 (MCA497R; Bio-Rad, Tokyo, Japan) and VECTASTAIN secondary antibodies (Vector Laboratories, Burlingame, CA, USA). To quantify the area stained by Oil Red O or F4/80, images of five random fields from each section were processed using Image J software (National Institute of Mental Health, Bethesda, MD, USA). Each value was expressed as a percentage of the total area of each positively stained section.

### 4.6. EdU Incorporation Assay

EdU cell proliferation assays were performed using the Click-iT™ Plus EdU Cell Proliferation Kit (C10639; Thermo Fisher Scientific, Waltham, MA, USA) according to the manufacturer’s instructions. Briefly, mice were intraperitoneally injected with EdU (100 μg/mouse) and, 24 h later, the dorsal lesion was collected and used to prepare a frozen block. The blocks were stained and images were taken using a confocal laser scanning microscope LSM710 (Carl Zeiss, Jena, Germany). To quantify EdU uptake, the number of EdU-positive cells in five locations in each section was counted and averaged.

### 4.7. Quantitative Real Time Polymerase Chain Reaction (RT–qPCR)

Total RNA was extracted from liver and skin tissues using the RNeasy^®^ Mini Kit (74106, QIAGEN, Hilden, Germany). RNA was reverse-transcribed using the SuperScript VILO cDNA Synthesis Kit (11754, Thermo Fisher Scientific, Waltham, MA, USA). Diluted cDNA was used as a template for quantifying relative mRNA concentrations. RT-qPCR was performed using Taqman Gene Expression master Mix (Thermo Fisher Scientific, Waltham, MA, USA), Taqman probes (Table S1), and the 7900HT Sequence Detection System (Thermo Fisher Scientific, Waltham, MA, USA). The primers used are listed in Table S1. Relative gene expression values were normalized to *B2M* for the liver and *Polr1a* for the skin, using the comparative Ct (threshold cycle) method.

### 4.8. RNA Sequence Analysis

RNA sequence analysis was conducted as previously described [21,22]. Briefly, sequencing was performed on an Illumina HiSeq 2500 platform in 75-base single-end mode. Illumina Casava 1.8.2 software (Illumina Inc, San Diego, CA, USA) was used for base calling. Raw reads were mapped to the mouse reference genome (mm10) using TopHat ver. 2.0.13 (Baltimore, MD, USA) [39] in combination with Bowtie2 ver. 2.2.3 (San Diego, CA, USA) [40] and SAMtools ver. 0.1.19 (San Diego, CA, USA) [41]. The number of fragments per kilobase of exons per million mapped fragments was calculated using Cufflinks ver. 2.2.143,44 (Seattle, WA, USA) [42] and was visualized as a heatmap. Pathway analyses were conducted using the STRING network tool. The raw data generated in this study are available in the Gene Expression Omnibus database (accession number GSE214837).

### 4.9. Statistical Analyses

The results are presented as mean ± SD. Pairwise comparisons were made using the two-tailed Student’s *t*-test. Multiple group comparisons were made using one-way ANOVA, followed by Tukey’s post hoc test. *p* < 0.05 was considered statistically significant.

## 5. Conclusions

The presence of oxysterol 7KC in the diet promoted IMQ-induced psoriasis-like dermatitis, mediated by the exacerbation of steatohepatitis, in mice. These findings suggest a novel strategy for treating steatohepatitis and psoriasis by reducing intestinal 7KC absorption. 

## Data Availability

The data presented in this study are openly available in the Gene Expression Omnibus database (accession number GSE214837).

## Supplementary Materials

The following supporting information can be downloaded at: https://www.mdpi.com/article/10.3390/ijms232415855/s1.

## References

[1] Lowes M.A., Bowcock A.M., Krueger J.G. (2007). Pathogenesis and therapy of psoriasis. Nature.

[2] Lowes M.A., Chamian F., Abello M.V., Fuentes-Duculan J., Lin S.L., Nussbaum R., Novitskaya I., Carbonaro H., Cardinale I., Kikuchi T. (2005). Increase in TNF-alpha and inducible nitric oxide synthase-expressing dendritic cells in psoriasis and reduction with efalizumab (anti-CD11a). Proc. Natl. Acad. Sci. USA.

[3] Madden S.K., Flanagan K.L., Jones G. (2020). How lifestyle factors and their associated pathogenetic mechanisms impact psoriasis. Clin. Nutr..

[4] Gelfand J.M., Neimann A.L., Shin D.B., Wang X., Margolis D.J., Troxel A.B. (2006). Risk of myocardial infarction in patients with psoriasis. JAMA.

[5] Takahashi H., Iizuka H. (2012). Psoriasis and metabolic syndrome. J. Dermatol..

[6] Kanemaru K., Matsuyuki A., Nakamura Y., Fukami K. (2015). Obesity exacerbates imiquimod-induced psoriasis-like epidermal hyperplasia and interleukin-17 and interleukin-22 production in mice. Exp. Dermatol..

[7] Gisondi P., Targher G., Zoppini G., Girolomoni G. (2009). Non-alcoholic fatty liver disease in patients with chronic plaque psoriasis. J. Hepatol..

[8] Miele L., Vallone S., Cefalo C., La Torre G., Di Stasi C., Vecchio F.M., D’Agostino M., Gabrieli M.L., Vero V., Biolato M. (2009). Prevalence, characteristics and severity of non-alcoholic fatty liver disease in patients with chronic plaque psoriasis. J. Hepatol..

[9] Wenk K.S., Arrington K.C., Ehrlich A. (2011). Psoriasis and non-alcoholic fatty liver disease. J. Eur. Acad. Dermatol. Venereol..

[10] Candia R., Ruiz A., Torres-Robles R., Chavez-Tapia N., Mendez-Sanchez N., Arrese M. (2015). Risk of non-alcoholic fatty liver disease in patients with psoriasis: A systematic review and meta-analysis. J. Eur. Acad. Dermatol. Venereol..

[11] Duell P.B., Welty F.K., Miller M., Chait A., Hammond G., Ahmad Z., Cohen D.E., Horton J.D., Pressman G.S., Toth P.P. (2022). Nonalcoholic Fatty Liver Disease and Cardiovascular Risk: A Scientific Statement from the American Heart Association. Arter. Thromb. Vasc. Biol..

[12] Hawkes J.E., Chan T.C., Krueger J.G. (2017). Psoriasis pathogenesis and the development of novel targeted immune therapies. J. Allergy Clin. Immunol..

[13] Tang Y., Bian Z., Zhao L., Liu Y., Liang S., Wang Q., Han X., Peng Y., Chen X., Shen L. (2011). Interleukin-17 exacerbates hepatic steatosis and inflammation in non-alcoholic fatty liver disease. Clin. Exp. Immunol..

[14] Vasseur P., Serres L., Jegou J.F., Pohin M., Delwail A., Petit-Paris I., Levillain P., Favot L., Samson M., Yssel H. (2016). High-Fat Diet-Induced IL-17A Exacerbates Psoriasiform Dermatitis in a Mouse Model of Steatohepatitis. Am. J. Pathol..

[15] Dainichi T., Kitoh A., Otsuka A., Nakajima S., Nomura T., Kaplan D.H., Kabashima K. (2018). The epithelial immune microenvironment (EIME) in atopic dermatitis and psoriasis. Nat. Immunol..

[16] Poli G., Leoni V., Biasi F., Canzoneri F., Risso D., Menta R. (2022). Oxysterols: From redox bench to industry. Redox Biol..

[17] Echarte M., Ansorena D., Astiasaran I. (2003). Consequences of microwave heating and frying on the lipid fraction of chicken and beef patties. J. Agric. Food Chem..

[18] Brown A.J., Jessup W. (1999). Oxysterols and atherosclerosis. Atherosclerosis.

[19] Hitsumoto T., Takahashi M., Iizuka T., Shirai K. (2009). Clinical significance of serum 7-ketocholesterol concentrations in the progression of coronary atherosclerosis. J. Atheroscler Thromb..

[20] Song J., Wang D., Chen H., Huang X., Zhong Y., Jiang N., Chen C., Xia M. (2017). Association of Plasma 7-Ketocholesterol with Cardiovascular Outcomes and Total Mortality in Patients with Coronary Artery Disease. Circ. Res..

[21] Chang J., Koseki M., Saga A., Kanno K., Higo T., Okuzaki D., Okada T., Inui H., Tanaka K., Asaji M. (2020). Dietary Oxysterol, 7-Ketocholesterol Accelerates Hepatic Lipid Accumulation and Macrophage Infiltration in Obese Mice. Front. Endocrinol..

[22] Kanno K., Koseki M., Chang J., Saga A., Inui H., Okada T., Tanaka K., Asaji M., Zhu Y., Ide S. (2022). Pemafibrate suppresses NLRP3 inflammasome activation in the liver and heart in a novel mouse model of steatohepatitis-related cardiomyopathy. Sci. Rep..

[23] Wilson N.J., Boniface K., Chan J.R., McKenzie B.S., Blumenschein W.M., Mattson J.D., Basham B., Smith K., Chen T., Morel F. (2007). Development, cytokine profile and function of human interleukin 17-producing helper T cells. Nat. Immunol..

[24] Terasaka N., Wang N., Yvan-Charvet L., Tall A.R. (2007). High-density lipoprotein protects macrophages from oxidized low-density lipoprotein-induced apoptosis by promoting efflux of 7-ketocholesterol via ABCG1. Proc. Natl. Acad. Sci. USA.

[25] Fredriksson T., Pettersson U. (1978). Severe psoriasis—Oral therapy with a new retinoid. Dermatologica.

[26] Van der Fits L., Mourits S., Voerman J.S., Kant M., Boon L., Laman J.D., Cornelissen F., Mus A.M., Florencia E., Prens E.P. (2009). Imiquimod-induced psoriasis-like skin inflammation in mice is mediated via the IL-23/IL-17 axis. J. Immunol..

[27] Yang L., Jin L., Ke Y., Fan X., Zhang T., Zhang C., Bian H., Wang G. (2018). E3 Ligase Trim21 Ubiquitylates and Stabilizes Keratin 17 to Induce STAT3 Activation in Psoriasis. J. Invest. Dermatol..

[28] Du Y., Jiang S., Cheng L., Liu J. (2020). JAK/STAT and VEGF/PAK1 signaling as emerging targets for topical treatment of psoriasis: A pilot study. Int. J. Clin. Exp. Pathol..

[29] Grabarek B., Krzaczyński J., Strzałka-Mrozik B., Wcisło-Dziadecka D., Gola J. (2019). The influence of ustekinumab on expression of STAT1, STAT3, STAT4, SOCS2, and IL17 in patients with psoriasis and in a control. Dermatol. Ther..

[30] Serviddio G., Bellanti F., Villani R., Tamborra R., Zerbinati C., Blonda M., Ciacciarelli M., Poli G., Vendemiale G., Iuliano L. (2016). Effects of dietary fatty acids and cholesterol excess on liver injury: A lipidomic approach. Redox Biol..

[31] Wang Y., Wang W., Wang N., Tall A.R., Tabas I. (2017). Mitochondrial Oxidative Stress Promotes Atherosclerosis and Neutrophil Extracellular Traps in Aged Mice. Arter. Thromb. Vasc. Biol..

[32] Anderson A., Campo A., Fulton E., Corwin A., Jerome W.G., O’Connor M.S. (2020). 7-Ketocholesterol in disease and aging. Redox Biol..

[33] Rau M., Schilling A.K., Meertens J., Hering I., Weiss J., Jurowich C., Kudlich T., Hermanns H.M., Bantel H., Beyersdorf N. (2016). Progression from Nonalcoholic Fatty Liver to Nonalcoholic Steatohepatitis Is Marked by a Higher Frequency of Th17 Cells in the Liver and an Increased Th17/Resting Regulatory T Cell Ratio in Peripheral Blood and in the Liver. J. Immunol..

[34] Li H.J., Wu N.L., Pu C.M., Hsiao C.Y., Chang D.C., Hung C.F. (2020). Chrysin alleviates imiquimod-induced psoriasis-like skin inflammation and reduces the release of CCL20 and antimicrobial peptides. Sci. Rep..

[35] Xie X., Zhang L., Lin Y., Wang Y., Liu W., Li X., Li P. (2017). Imiquimod induced ApoE-deficient mice might be a composite animal model for the study of psoriasis and dyslipideamia comorbidity. J. Dermatol. Sci..

[36] Vasseur P., Pohin M., Jégou J.F., Favot L., Venisse N., Mcheik J., Morel F., Lecron J.C., Silvain C. (2018). Liver fibrosis is associated with cutaneous inflammation in the imiquimod-induced murine model of psoriasiform dermatitis. Br. J. Dermatol..

[37] Yokogawa M., Takaishi M., Nakajima K., Kamijima R., Fujimoto C., Kataoka S., Terada Y., Sano S. (2014). Epicutaneous application of toll-like receptor 7 agonists leads to systemic autoimmunity in wild-type mice: A new model of systemic Lupus erythematosus. Arthritis Rheumatol..

[38] Sano S., Chan K.S., Carbajal S., Clifford J., Peavey M., Kiguchi K., Itami S., Nickoloff B.J., DiGiovanni J. (2005). Stat3 links activated keratinocytes and immunocytes required for development of psoriasis in a novel transgenic mouse model. Nat. Med..

[39] Kim D., Pertea G., Trapnell C., Pimentel H., Kelley R., Salzberg S.L. (2013). TopHat2: Accurate alignment of transcriptomes in the presence of insertions, deletions and gene fusions. Genome Biol..

[40] Langmead B., Salzberg S.L. (2012). Fast gapped-read alignment with Bowtie 2. Nat. Methods.

[41] Bonfield J.K., Marshall J., Danecek P., Li H., Ohan V., Whitwham A., Keane T., Davies R.M. (2021). HTSlib: C library for reading/writing high-throughput sequencing data. Gigascience.

[42] Trapnell C., Williams B.A., Pertea G., Mortazavi A., Kwan G., van Baren M.J., Salzberg S.L., Wold B.J., Pachter L. (2010). Transcript assembly and quantification by RNA-Seq reveals unannotated transcripts and isoform switching during cell differentiation. Nat. Biotechnol..

