# Supplementary material for: A Dietary Oxysterol, 7-Ketocholesterol, Exacerbates Imiquimod-Induced Psoriasis-like Dermatitis in Steatohepatitic Mice
Source: Int J Mol Sci. 2022 Dec 13;23(24):15855. doi: 10.3390/ijms232415855 (PMC9785382; doi:10.3390/ijms232415855)
Supplement: Supplementary file 1 [file ijms-23-15855-s001.zip › ijms-1966383-supplementary.pdf]

International Journal of
Molecular Sciences

MDPI

Supplement Table S1. qPCR probe list.

probe	
Tnfa	Mm00443258_m1
Tgfb1	Mm01178820_m1
Il1b	Mm00434228_m1
Il6	Mm00446190_m1
Il12b	Mm01288989_m1
Il23a	Mm00518984_m1
Il17a	Mm00439618_m1
Il17c	Mm00521397_m1
Il17f	Mm00521423_m1
Il22	Mm01226722_m1
Nlrp3	Mm00840904_m1
Ccl2	Mm00441242_m1
Krt6	Mm00833464_g1
Krt16	Mm01306670_g1
Krt17	Mm00495207_m1
B2m	Mm00437762_m1
Polr2a	Mm00839493_m1

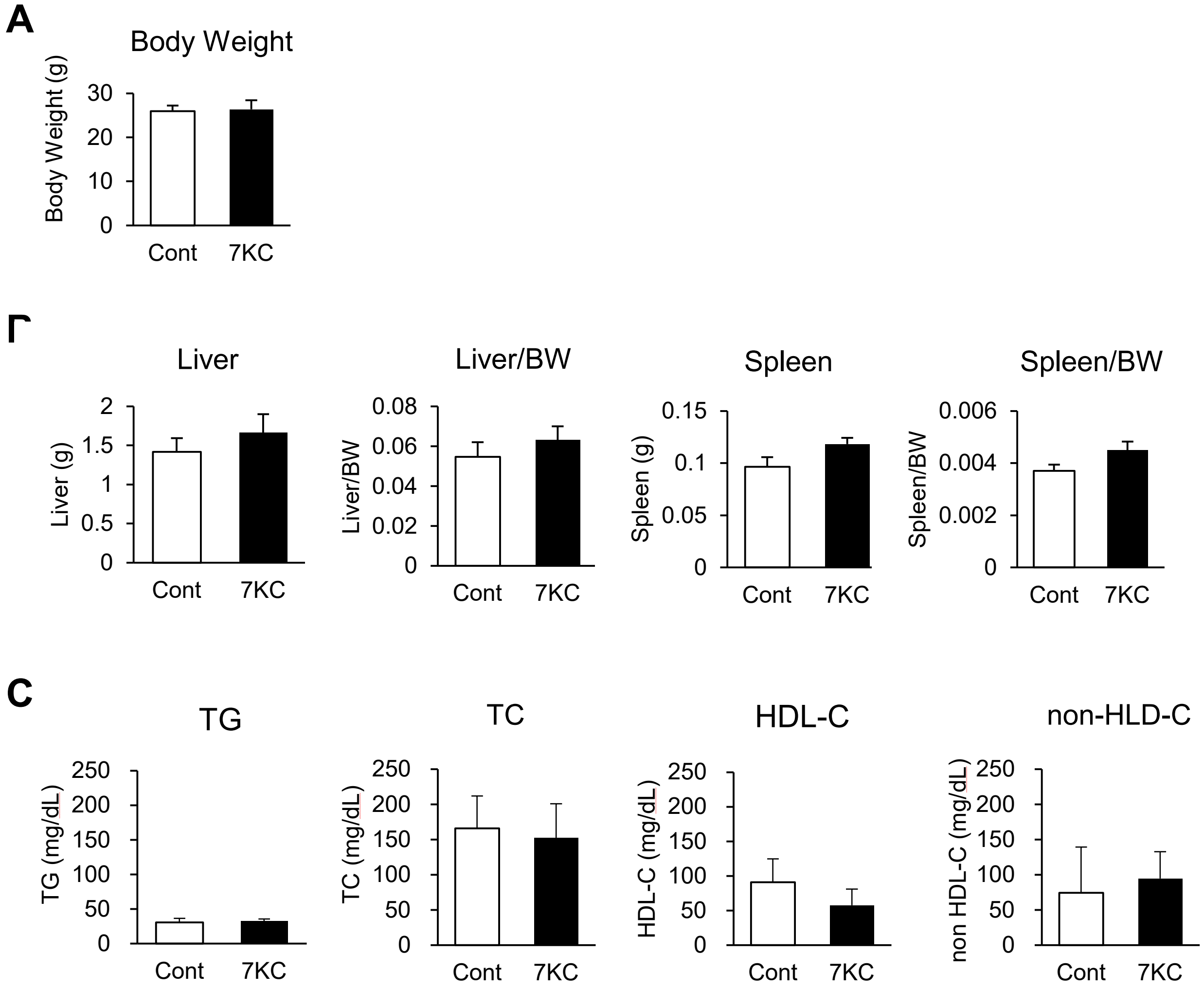

Figure S1. Body weight, Liver and spleen weights, and serum lipids profile on Day 0. Mice were fed a NASH or 7KC diet for three weeks, and samples were collected on Day 0. (**A**) Body weight. (**B**) Liver and spleen weights. (**C**) Serum lipid profile Results are presented as mean ± SD, and *p*-values were calculated using Student's *t*-test. * $p < 0.05$, Control vs. 7KC. $n = 6$, Control; $n = 6$, 7KC.

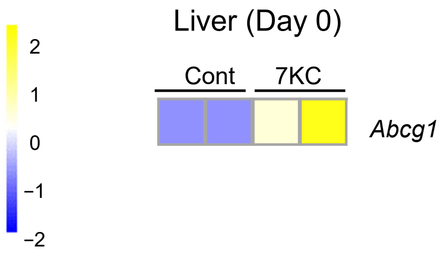

Figure S2. RNA sequence analysis of Abcg1 in liver on Day 0.
